# Modeling perceptions of climatic risk in crop production

**DOI:** 10.1371/journal.pone.0181954

**Published:** 2017-08-01

**Authors:** Evelyn Reinmuth, Phillip Parker, Joachim Aurbacher, Petra Högy, Stephan Dabbert

**Affiliations:** 1 Institute of Farm Management, Section Production Theory and Resource Economics, Universität Hohenheim, Stuttgart, Germany; 2 Institute of Farm and Agribusiness Management, Justus-Liebig-University Gießen, Gießen, Germany; 3 Institute of Landscape and Plant Ecology, Universität Hohenheim, Stuttgart, Germany; University of Reading, UNITED KINGDOM

## Abstract

In agricultural production, land-use decisions are components of economic planning that result in the strategic allocation of fields. Climate variability represents an uncertainty factor in crop production. Considering yield impact, climatic influence is perceived during and evaluated at the end of crop production cycles. In practice, this information is then incorporated into planning for the upcoming season. This process contributes to attitudes toward climate-induced risk in crop production. In the literature, however, the subjective valuation of risk is modeled as a risk attitude toward variations in (monetary) outcomes. Consequently, climatic influence may be obscured by political and market influences so that risk perceptions during the production process are neglected. We present a utility concept that allows the inclusion of annual risk scores based on mid-season risk perceptions that are incorporated into field-planning decisions. This approach is exemplified and implemented for winter wheat production in the Kraichgau, a region in Southwest Germany, using the integrated bio-economic simulation model FarmActor and empirical data from the region. Survey results indicate that a profitability threshold for this crop, the level of “still-good yield” (*sgy*), is 69 dt ha-1 (regional mean Kraichgau sample) for a given season. This threshold governs the monitoring process and risk estimators. We tested the modeled estimators against simulation results using ten projected future weather time series for winter wheat production. The mid-season estimators generally proved to be effective. This approach can be used to improve the modeling of planning decisions by providing a more comprehensive evaluation of field-crop response to climatic changes from an economic risk point of view. The methodology further provides economic insight in an agrometeorological context where prices for crops or inputs are lacking, but farmer attitudes toward risk should still be included in the analysis.

## Introduction

Climate change contributes to the evolution of agricultural landscapes and is also subject to a feedback loop as changes in land use, especially conversion to cropland, drives processes such as the global carbon cycle that have been linked to changes in climatic conditions [1]. Variabilities in the biophysical processes in agricultural production have been found to be closely associated with climate [2–6]. The Intergovernmental Panel on Climate Change (IPCC) [7] stated in its Fifth Assessment Report that climate change is expected to amplify existing climatic risks. For crop farmers, this can represent a challenge in terms of the optimal allocation of land under their management [8]. The manager’s job is to choose crops suited to the environment in their location [9], subject to stipulations including risk aversion [10]. Strategic crop selection and rotation are a cornerstone of successful agricultural enterprises [11]. Studies of agricultural production systems must account for managerial strategies that are based on dynamic environmental and economic conditions. Climate change impact assessment usually treats land use as a planning decision. The climatic influence is regarded as a source of gross-margin variation, which makes planning more difficult, and it is therefore considered to be a source of risk and uncertainty [12]. Available methodological approaches have been found to either over- or underestimate the rate of adaptation by farmers to climatic impacts [13–16].

Furthermore, economic drivers, such as commodity prices or agricultural policy, have been found to dominate climatic influences at the gross-margin level, particularly in terms of the speed of farmer response to stimuli [17, 18], through planning decisions. The challenge lies in the isolation of the climatic influence in such risk analysis [14, 19]. The aim of the present work is to exemplify a more comprehensive methodology for incorporating risk, in particular climate-induced risk, into agricultural simulation. This approach should contribute to the ongoing pursuit of more robust system representation to enable more reliable predictive capacity.

## Materials and methods

Currently used models show how climate change can be expected to induce transition processes in agricultural land use, for example the Integrated Land-use Model (ILM) [20] and MPMAS [21]. Although they are coupled with complex biophysical models (EPIC [22] and Expert-N [23], respectively), these models consider only the final outcome of the production process, such as yield. Valuable information produced throughout the simulated growing cycle is ignored for lack of a realistic methodology to incorporate it into the representation of agricultural strategy.

Furthermore, in reality, farmers continuously evaluate crop development on their fields, even when no activities are pending. At certain intervals throughout the growing season, farmers base their estimates regarding eventual production outcomes on an evaluation of the current crop development stage [24]. This process is accompanied by an automatic perception of the crop’s response to the season’s climate at a certain location. This evaluative monitoring goes beyond a pure input efficiency analysis that accounts for (seasonal) variability in agricultural production, as has been investigated [24–28]. Over time, the aggregation of these evaluations shape a farm manager’s cumulative perception of the climate-induced variability of a crop in the form of a perceived response pattern. This knowledge is, in practice, incorporated into planning decisions, and can be used to set crop yield within the context of the respective production processes while including how climate affects production efficiency.

Thus, the primary hypothesis here is that incorporating inter-temporal evaluations of agro-economic processes in planning decisions will cultivate a more thorough understanding of the impact of climatic changes on land-use (planning) decisions and, thus, of how agricultural landscapes evolve over time.

Bio-economic simulation models with dynamic module integration (e.g., crop-soil-management-weather-prices), operating at adequate spatio-temporal resolution [29], offer the possibility of investigating different processes and dynamics of decision-making over time while being representative of the real world [30]. Mid-season risk perceptions can only be modeled at fine (e.g., daily, at least sub-annual) resolution [14]. Few bio-economic models operate at an appropriate temporal resolution to allow the study of farmers’ perception processes during the growing season (APSIM [31] and FarmActor [32]).

The APSIM model [31], is able to mimic production process decisions at daily resolution and at the field level. Crop management decisions can be made according to weather, plant and soil conditions. In the APSIM model, a trigger mechanism is used to adjust tactical decision-making in the context of response farming [33]. The FarmActor model, as coupled to the Expert-N crop model [23, 34–37], incorporates all the functionality of the APSIM model in terms of daily management in response to soil-crop-weather dynamics [32]. FarmActor is further able to adapt simulated crop management in response to long-term climatic influences through model-endogenous mechanisms. It therefore resonates with actual agricultural activity at multiple scales (daily, yearly, decadal, etc.) of the managerial perspective and, thus, at scales where strategy can be implemented. Both models are designed to explore the optimization of production routines and the planning process in an agro-meteorological context.

The FarmActor model’s environmental condition triggers that govern field management decision-making are designed to mimic agronomic reasoning such that it can accurately reproduce empirically observed farmer behavior, particularly the timing of sowing and harvest [38]. Observed behavior, in a soil-weather context, is assumed to be in pursuit of profit maximization in response to embedded production risks. The timing of field management actions provides a traceable link between farm strategy and eventual yields. The extraction of economic reasoning that drives observed statistics is tenuous [39], whereas in bio-economic simulation, trigger settings provide a transparent mechanism to quantify the system components that steer productivity.

By definition, bio-economic simulation models go beyond agronomic aspects and are designed to use the coupling of model components to integrate agronomic aspects into economic analysis. This functionality is the key to integrated process evaluations in economic planning decisions but has yet to be well developed for APSIM and FarmActor.

### Data and study region

Data were obtained empirically as part of a structured survey on risk and decision-making in agriculture that was conducted in the Kraichgau study region in Southwest Germany (~1,400 km^2^) [32]. The Kraichgau is, from an agricultural point of view, a fertile region with intensive agricultural activity on mostly loess-rich soils and a relatively homogenous agricultural landscape [40, 41]. Elevations are between 100 and 400 m asl with a landscape defined by moderate slopes. The mean annual precipitation ranges from 730 to 830 mm and has a mean annual temperature of 9 to 10°C.

The farmers were informed through an introductory cover letter about the content and purpose of the questionnaire and the planned use of their data. They were further assured that all procured data would be evaluated with the safeguard of anonymity and for scientific purposes only within the framework of the research group and that their personal information would be treated confidentially. As an incentive to increase the response rate, there was also the possibility of participating in a lottery drawing to win one of 20 vouchers for a farm supply company; each voucher had a value of 30 EUR. The response rate was 23.4%, or 91 completed questionnaires.

No ethics committee or institutional review board was contacted because it was not compulsory at the time. The questionnaires were read by all members of the research group and were approved by all responsible project leaders before they were sent out. The empirical work was subsequently approved by the Ethics Committee of the University of Hohenheim, 70599 Stuttgart.

### Empirical approach

Mid-season evaluation is conducted to assess variations in the crop development process [42] and, when significant, can provide expectations regarding eventual yield levels. Preferential outcomes during the season or at harvest are constructed after consideration of an immense number of possible options that a decision-maker faces, many of which are similar in nature [43]. Therefore, the agroeconomic focus should be on thresholds as a component of wealth dynamics, as suggested by Just et al. [44] and Antle [25], to scale preferences properly. Risk bearing, which is also a scalar, is related to a decision-maker’s willingness to accept (*wta*) outcome fluctuations [45]. Willingness to accept is a downside-risk measure because it focuses on incurred losses and individual assessment of such losses [46]. Because aspects of preferences and perceived risks are constructed according to local conditions, there is a need to establish a context within which an economic subject acts.

This need led to the execution of the farmer survey in the Kraichgau region. Farmers were asked to give a subjective evaluation of crop observations according to their experience with all crops in their production branch (question 1.11 of S1 Questionnaire). Downside-risk attitude was elicited in a crop-wise manner because of the highly resolved decision-making component in the FarmActor model that plans the allocation of crops for each field of a farm individually and, thus, mid-season observations are modeled crop-specific.

The willingness to accept is defined in physical units as follows: *py–sgy = wta*, where *py* = peak yield and *sgy* = (farmer-defined) “still-good yield”. The *sgy* level was set as the lower boundary of a farmer’s acceptable level of fluctuation. All values above the *sgy* provide utility to a farmer in an economic sense. Below the *sgy*, the values represent the production outcome levels that might imply agronomic non-profitability according to farmer experience; more specifically, they imply economic losses and thus represent risk.

To compare different *sgy* statements, a definition for a sensitivity type in this context is provided that uses the third elicited point of a farmers’ crop yield distribution, i.e., the average yield (avy_farm_). Sensitivity types are similar to risk aversion types, as suggested by Arrow [47] and Pratt [48] to account for farmers having a different subjective acceptance of risk [49] and are part of the methodological concept to be presented.

#### Sensitivity types

The average yield (*avy*_*farm*_) determines the location of *sgy* in the yield distribution and allows for the identification of comparative performance between farms for a given crop. We can argue that when *avy*_*farm*_ > *avy*_*region*_ for a given crop, the farmer’s yield distribution and the processes underlying the result are likely to be favorably skewed. This reasoning is based on the assumption that climatic influences are comparable throughout the relatively uniform Kraichgau study region and production conditions, such as the availability of inputs are assumed to be relatively homogenous throughout this region.

If a farmer declares *sgy* to exceed *avy*_farm_, despite *avy*_farm_ being higher than *avy*_region_, the farmer can be considered to be more sensitive to yield for a particular crop as opposed to a farmer that declares his *sgy* below *avy*_farm_.

A sensitivity type in this context is thus identified by two conditions with regard to a given crop: first, determine if *sgy* is above or below *avy*_farm_; second, compare the individual average performance of a crop at the farm level *avy*_farm_ to the individual *avy*_farm_.

### Empirical results

Despite *avy*_farm_>*avy*_region_, only 13, or 17.81% (n = 73), of the farmers in the Kraichgau sample declared their *sgy* above *avy*_farm_ and could thus be identified as being highly sensitive with regard to fluctuations of one or more crops produced on their farm. Due to the low sample size and strong sample heterogeneity, neither regression nor cluster analysis yielded significant results when attempting to categorize the sampled Kraichgau farmers into typical groups according to age, experience and stated farm characteristics. However, this particular information is not necessary to introduce the methodological approach.

Descriptive statistics for the three subjective values (peak, still-good and average yield) as reported by questionnaire respondents regarding winter wheat in the Kraichgau are displayed in Table 1. Winter wheat results are presented because of their economic importance and their use to exemplify the methodological approach. Of 91 returned questionnaires, 82 incorporated information related to this study.

**Table 1:** Empirical results for winter wheat from the Kraichgau sample.

	Mean	Min	Max	Std. Dev.	Skewness	Variance	Var. Coef.	n
**Peak yield (dt/ha)**	87.78	65	116	10.36	-0.17	107.43	0.12	81
**Still-good yield (dt/ha)**	69.17	40	90	10.05	-0.70	100.92	0.15	78
**Average yield (dt/ha)**	74.44	50	90	8.43	-1.00	70.98	0.11	80

Note: Min = Minimum, Max = Maximum, Std. Dev. = Standard Deviation; Var. Coef. = Variation Coefficient. Different sample sizes are related to various levels of questionnaire completion. Source: Own survey (2013), question 1.11 of S1 Questionnaire.

For the Kraichgau sample, the average reported *sgy* is below the mean reported *avy*, which is near the five-year (2008–2012) mean yield of 71.4 dt/ha for winter wheat across the four districts (Landkreise) that comprise the study region [50].

The empirically elicited *sgy* threshold was then translated for mid-seasonal process observations using the FarmActor model based on the following utility concept. The *sgy* threshold is used to scale perceived variance during production from an economic perspective [14].

### Utility concept

The introduction of utility is necessary and a useful measure for economic evaluations during the season and to ensure compatibility with economic reasoning at the level of planning [51] in the simulation model.

Thus, at sequential observation points (OPs), which represent specific crop management or monitoring actions, the modeled farmer evaluates crop development, soil water content and soil temperature, as driven by soil-plant-climate interactions, using simulation model output parameters, namely, the set that describes the current status, with *i* ∈ (1,…,*n*) as the total number of possible (crop-specific) observation parameters. The evaluation of each observed parameter value is assessed with a utility score *α*_*i,c*_ at each OP (c stands for crop).

Each parameter has an assigned acceptance range that triggers the monitoring procedure in the model. Anticipated economic profitability and thus an absence of risk (*α*_*i,c*_ = 0) are implied when the observation parameter lies inside a predefined acceptance range; yield levels above the *sgy* threshold are expected based on the inter-temporal observation. A utility score of 1 results during a “risky” season when an observed parameter value lies ***outside*** a predefined acceptance range that is associated with crop profitability. The observational procedure is independent of simulated management. It is only used to accumulate perceptions of the climatic influence dependent on the *sgy* threshold.

The utility risk score at an individual OP for a crop is represented as the sum of the utilities of all observation point parameters:

$$\alpha_{t,c}=\sum_{i=1}^{n}\alpha_{i,c}\;\mathrm{for}\;\mathrm{all}\;t\;\mathrm{and}\;c$$ (1)

where *t* is an OP at a certain point in time with *t* ∈ (1,…*m*) within the growing process of crop *c*. The season’s accumulated utility values of all OP’s are summed after harvest to obtain *α*_*T,c*_, the total utility score, or ARS, of a production year for a given crop as the result of the intermediate rankings. This relation can be written as

$$ARS=\alpha_{T,c}=\sum_{t=1}^{m}\alpha_{t,c}\;\mathrm{for}\;\mathrm{all}\;c.$$ (2)

**Fig 1:**
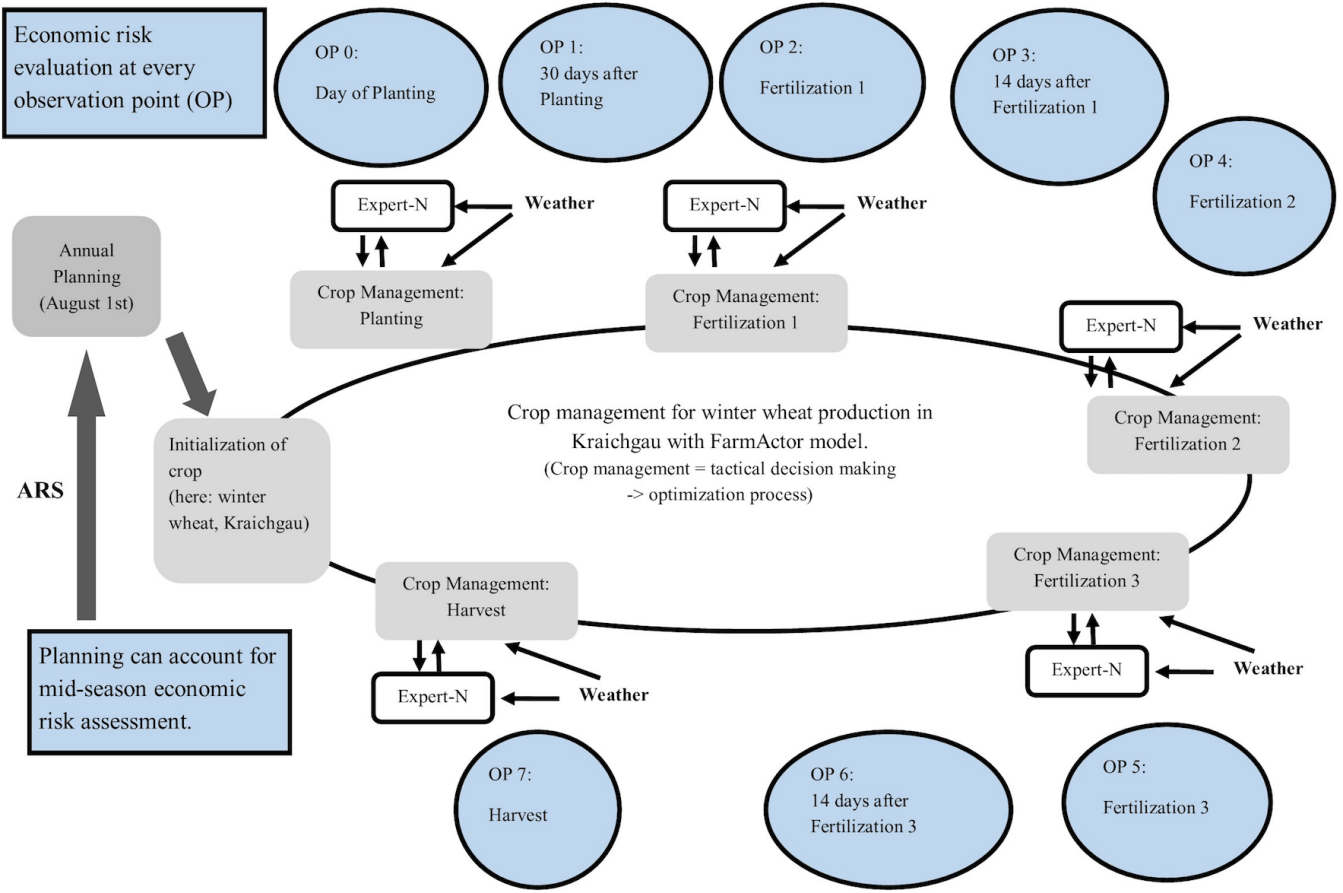
Economic risk evaluation via mid-season assessment. Risk perceptions are documented at eight OPs, 0 to 7, in the FarmActor model. The example of winter wheat production in the Kraichgau is shown. Each observation is conducted parallel to wheat production. Source: Adapted from [32].

Fig 1 exemplifies the ARS concept for winter wheat production in the Kraichgau. The timing for the OPs is inferred from the FarmActor model’s list of field management actions for winter wheat production. The management procedure in the simulation model has been previously calibrated for Kraichgau (see, [32]). The chosen OPs represent one possible option and can be adjusted for other crops.

Most importantly and to understand the evaluative monitoring, an ARS score and its components are not used to govern the inter-temporal optimization process but, rather, as an instrument for risk assessment and selecting relevant information regarding the climatic influence. Therefore, each *α*_*t,c*_ identifies the composite risk (across observed conditions) for a given observation point that becomes part of an overall assessment at the end of the year.

There is no further weighting of inter-temporal utility coefficients because the utility of a value is only revealed *ex post* [12, 52] despite the fact that the utility regarding sequential observations has been referred to as the “utility of the moment” [53], indicating that it is only valid for a short period of time until the situation reveals new information to be processed [54]. The state of available information is the factor that changes with subsequent crop development and observation, while the uncertainty regarding further development remains the same [24].

#### Simulations

Before the simulation model is able to assess climatic impacts, based on the ARS methodology, the model must be provided with information about what level of mid-season variability is acceptable. The ARS methodology is exemplified using the empirically elicited average *sgy* from the Kraichgau sample, 69.17 dt/ha (Table 1), to scale the boundaries of the acceptance ranges in the simulation model.

Acceptance ranges for inter-temporal crop conditions embody the experience of a farmer in crop production. This experience pertains to a certain range of tolerable fluctuations in growth patterns from a downside risk perspective at a given point in time during the growing season. To initialize these ranges, it was necessary to simulate a reasonable number of “historical” growing seasons to accumulate an experience pool for the simulated farmer.

To create a knowledge pool, FarmActor’s crop management routine was overridden in order to compile a comprehensive set of simulated agro-ecological states (crop biomass, soil moisture, etc.) arrived at through what-if scenarios using an array of day-of-year (DOY) starting points (sowing dates). A standard production procedure was used for these model runs, and input levels for fertilization were kept constant. All wheat-specific actions, apart from planting (three fertilization applications, harvesting, and preparation of fields; Fig 1) were governed by their existing weather and plant-growth-dependent trigger arrays [32] and [38]. A range of plausible starting points for winter wheat growth processes for the Kraichgau region extends from DOY 283 to 324 (early-mid October to mid-late November) [38]. The later the simulated DOY for sowing, through the associated growth-process simulation, the less likely the yields will be acceptable or greater than *sgy*, thus justifying the maximum in the sowing DOY chosen for simulation. For each of the 46 DOY, a time series of 30 years (1983–2013) was modeled. This produced “historical” growth processes for each weather year and resulted in 1,380 permutations of the annual growth processes for winter wheat.

Data from the modeled growth processes were then used to initialize the acceptance ranges of all observation points used to monitor the growth processes. A season’s first observation point is the sowing date, OP 0 (see Fig 1). The acceptance range for OP 0 (sowing) is described by the relative frequency in which a DOY led to a yield ≥ *sgy (*average *sgy* for the Kraichgau region 69.17 dt/ha (Table 1)) throughout the historical time frame. This value is the ratio of desired to total yield outcomes compiled over all simulation years (n = 30) and stems from that particular sowing DOY. The higher the relative success frequency of a DOY, the more often a yield ≥ *sgy* is observable in the 30-year times series with this DOY as a starting point, and consequently, the more suitable a DOY is for sowing.

For crop surveillance after sowing, at OP 1 through 7, ten observable field parameters are used for monitoring to give a complete picture of crop response to external influences: *aboveground biomass* and *generative biomass* (kg/ha); *leaf area index* (LAI); *crop development stage* (BBCH) (BBCH-scale [55]); *soil water content* at 20, 50 and 120 cm depths (% volume); and *soil temperature* at 5, 20 and 50 cm depths (°C). The crop development stage is used indirectly; it is incorporated into the decision triggers of the simulation management routine to ensure that observations each year are made at a comparable stage of crop development (compare [32]).

Mid-season indicators of forthcoming yields, or OP 1 to 7, are based on simulated growth processes, however, not all modeled values for a given parameter could serve as a reference for an economically interpretable observation. The base of 1,380 data points for each OP parameter was reduced to those in which the corresponding final yield did not fall below the downside-risk threshold, i.e., the average *sgy* for the Kraichgau region, 69.17 dt/ha (Table 1). The results represent the farm actor’s “knowledge” that has accrued over a 30-year time-span. These data provide a simulated knowledge base to generate expectations about crop response to seasonal variability, with reference to a yield benchmark, which implies agronomic utility.

Because the states of physical growth processes are compiled as observational data, it is important to determine acceptance ranges that cover a wide range of outcomes to prevent the model from being too sensitive to fluctuations. When plotting the data with boxplots, the limits for each observation point parameter were set at the whiskers of selected point-specific outcomes for a given parameter. The outliers represent a result of rare combinations of plant response and environmental conditions that still resulted in a yield ≥ *sgy* and were thus excluded. The outliers are data points that lie below the first quartile minus 1.5 times the inter quartile range (x_0.25_−1.5 IQR) or above the third quartile plus 1.5 times the inter quartile range (x_0.75_ + 1.5 IQR). Whiskers are assigned to the next inner data points, which are not considered outliers. Boxplots were calculated with STATA [56]. We examined the result of the data extraction further to test whether it made any difference in our acceptance ranges when all 1,380 data points were plotted. The acceptance ranges were consequently much greater in both directions.

#### Acceptance ranges

Table 2 gives an overview of all acceptance ranges we obtained from extracting simulation data for all OPs in winter wheat production in the Kraichgau using the FarmActor model.

**Table 2:** Observation trigger ranges for winter wheat production in the Kraichgau region for the production years 1983–2013 and 10 observation parameters throughout 7 observation points; average regional *sgy* level: 69.17 dt/ha (Table 1); weather data from Eppingen station (see details [32]).

OP 0: Planting (day-of-year)	Min	280									
	Max	313									
		Leaf Area Index	Soil Water at				Soil Temperature at			Above Ground Biomass	BBCH
			30 cm	60 cm	90 cm	120 cm	5 cm	10 cm	50 cm		
**OP1: 30 days after** **planting**	Min	0.07	28.5	26.62	25.64	26.7	-6.01	-4.58	-0.18	39.27	8.06
	Max	0.77	34	35.58	35.02	32.63	12.64	11.86	10.33	302.52	13.58
**OP2: Fertilization 1**	Min	1.32	19.25	21.88	23.53	25.78	4.34	5.26	5.72	913.18	25
	Max	4.17	34.78	33.21	32.03	32.14	19.58	17.88	13.06	2744.6	25.81
**OP3: 14 days after** **fertilization 1**	Min	2.54	21.67	20.97	22.34	24.62	6.65	6.47	7.04	1709.2	27.05
	Max	6.05	36.09	33.39	31.57	31.15	20.46	19.69	14.75	4126.6	33.74
**OP4:** **Fertilization 2**	Min	2.57	21.67	21.98	22.23	24.57	7.53	7.72	8.72	1695.8	30
	Max	6.84	35.81	32.63	31.37	30.03	20.43	19.33	14.48	4690	32.88
**OP5:** **Fertilization 3**	Min	3.62	18.31	17.43	19.8	23.4	8.66	8.96	9.16	3404.4	39
	Max	7.25	37.28	35.63	32.05	29.12	21.23	20.31	15.5	6505	42.83
**OP6: 14 days after** **fertilization 3**	Min	3.84	16.12	15.2	17	21.06	10.09	10.05	10.41	6380.1	52.14
	Max	6.88	40.12	32.4	30.26	30.22	22.42	21.74	17.51	9631.9	61.26
**OP7: Harvest**	Min	1.17	10.87	11.13	11.72	16.27	13.64	13.54	13.1	7343.6^1^	90.7
	Max	2.12	32.99	31.01	27.16	27.63	21.67	20.56	18.91	8803^1^	92.7

Note

^1^ At **harvest,** the parameter observed is **generative (grain) biomass (kg/ha)**. The minimum and maximum values are respective of the lower and upper boundaries of an acceptance range for an observation parameter. Mid-season observations are based on aboveground biomass (dt FM/ha)—for example, at OP 2 (Fertilization 1). This measurement for winter wheat was usually between 913.18 and 2,744.6 in weather/production years that resulted in a yield ≥ *sgy* of 69.17 dt/ha. Thus, the observable biomass during a given year between these two values can still be considered as risk-neutral fluctuation or as not affecting downside risk in terms of economic loss expressed in yield levels.

Acceptance ranges represent tolerable fluctuation during the growth process from a downside-risk point of view. The values between the boundaries are not ranges that indicate the optimal state. The upper boundaries of the acceptance ranges, therefore, can be more than twice the value of the lower boundary, especially in the earlier observation point ranges. The later the observation point is, the closer the boundary values are. After initialization, the attained acceptance ranges with farmers’ revealed preferences for yield fluctuations are used to test and exemplify the approach. Thus, in the next section, the knowledge pool based on past climatic impacts is used to test how changes in future climatic conditions can be perceived by the model farmer using the ARS methodology.

#### Application to future climate

Future simulations for winter wheat production in the Kraichgau were performed using the coupled FarmActor-Expert-N models forced with simulated future climate in the form of daily weather parameters in ten realizations (25002–00 and 25002–99) of WETTREG 2010 future scenarios [57], corresponding to the German Weather Service [58] weather station at Eppingen (Kraichgau study site) and based on the IPCC scenario A1B [59]. For each WETTREG future realization, the model simulated a 20-year (2014–2034) time series for winter wheat with only one standard production procedure each year on a representative field [32]. FarmActor’s management routine was not manipulated in this case. These ten time series, each with 20 years of future growth processes, were then used to comprehensively test the methodological approach. All growth processes were evaluated with the ARS mechanism by comparing them to their acceptance range at each observation point (OP 0–7) to conduct a comparative analysis and investigate the performance of the proposed approach under diverse climatic conditions.

As described above, observations outside the acceptance ranges are assigned a binary utility value of 1 (0 otherwise). At the end of each year, from evaluating all observations made during the production season, the ARS is compiled. With 10 observation parameters and 7 observation points (OP 1 to OP 7) plus OP 0 for planting, a maximum score of 71 is possible for a year. OP 0 is related to the desirability of a given DOY for planting. If its relative frequency of yields ≥ *sgy* is less than 0.5, that is to say, if more than half of the time the harvest is disappointing, this observation is assigned a value of 1. Thus, if planting is forced to a day (through weather-dependent trigger conditions) that has a relative frequency value less than 0.5, then OP 0 is assigned a value of 1 for that production year.

#### Results of future climate simulations

The results of this investigation are displayed in a comparative static context in Table 3, in which the results are further refined into a mean-standard deviation approach.

**Table 3:** Simulated yield statistics for over 20 years, 2014–2033, using 10 WETTREG future realizations for Eppingen weather station.

WETTREG future weather realization	00	11	22	33	44	55	66	77	88	99
**Mean yield (dt/ha)**	74.54	68.61	70.31	68.64	72.39	72.39	65.18	65.15	71.41	61.77
**Standard deviation (dt/ha)**	3.50	16.26	16.97	16.44	4.72	4.72	21.97	21.97	5.57	26.25
**Skewness**	0.31	-4.07	-3.88	-3.90	-1.94	-1.94	-2.76	-2.76	0.40	-2.04
**Variation coefficient**	0.05	0.24	0.24	0.24	0.07	0.07	0.34	0.34	0.08	0.43
**Rel. Frequency y** **≥** **sgy**	0.95	0.7	0.75	0.7	0.85	0.85	0.7	0.65	0.7	0.7
**Mean ARS score**	4	4.05	5	5.2	3.9	4.9	3.95	5.15	2.45	5.95
**Std. dev. ARS score**	3.70	3.76	4.22	4.10	3.16	4.52	4.61	4.11	2.01	7.90

Note: All results have been obtained from the FarmActor standard production procedure for winter wheat in Kraichgau (see [32, 38]). Source: Own calculations.

The mean-standard deviation analysis provides an overview of how each of the ten future WETTREG weather realizations affects winter wheat production based on a standard production procedure. All ten realizations were used to avoid any bias in a single realization based on their statistical nature and to examine a range of possible outcomes using the *sgy* approach.

At a *sgy* level of 69.17 dt/ha, the results for the WETTREG-forced winter wheat production in the Kraichgau can be interpreted from a risk point of view as follows. Realizations 44 and 55 show the second lowest yield standard deviation (4.72 dt/ha, Table 3) for all future weather years. The standard deviation of ARS scores for the same realizations is noticeably higher than its counterparts. For realization 55, the standard deviation of all ARS scores is the third highest, although the yield standard deviation is the second lowest in the mean variance comparison. The relative frequency is also relatively favorable, at 0.85 (Table 3) for realizations 44 and 55. Thus, for 85% of the 20-year yield time series, a yield ≥ *sgy* of 69.17 dt/ha could be achieved. This finding represents favorable skewness for a decision-maker who sets his economic profitability yield threshold at this level. However, when taking a farmer´s mid-season assessment into account, those future production years are characterized by relatively large deviations from the modeled acceptance ranges. This result gives an indication that farmers often experience anomalous winter wheat performance sometime during the season, even in favorable years with a yield ≥ *sgy*.

The additional information provided here for the analysis is that a farmer’s mid-season observations are often ambiguous regarding what yield can be expected at a harvest [25, 45].

A different picture is drawn with realization 99. For such a projection of future climatic conditions, a mean-variance analysis at yield level illustrates the same issue as the ARS methodology, in which the highest mean of ARS scores corresponds to the highest standard deviation of ARS scores. The mean yield is among the lowest, and the standard deviation is the highest. Low yields that are traceable to a problematic climate at the 69.17 dt/ha *sgy* level show how mean-variance analysis at a yield level leads to the same conclusions as the ARS methodology. Farmers’ knowledge of crop-response patterns to climatic influences holds true and leads to accurate expectations regarding their yields.

The picture drawn with realization 11 in Table 3 is most interesting. The mean-variance analysis shows a completely different picture than the ARS methodology. This realization led to one of the highest standard deviations (16.26 dt/ha) in yields and a relatively low mean yield (68.61 dt/ha) (Table 3), close to the average *sgy* level for the Kraichgau region (69.17 dt/ha, Table 1). However, the mean ARS score is at 4.05 (Table 3), which is not among the highest scores achieved over all future climate realizations. This finding indicates that conditions were not unfavorable throughout the studied growing season but rather single events were responsible for the production outcomes in this time series of winter wheat yields.

## Discussion

A modeling approach that can account for climatic stress that is normally hidden from economic analysis can more thoroughly examine climatic impacts and provide more information.

The results of a first application of the ARS methodology with mid-season estimators show generally effective outcomes. Inter-temporal observations can provide better information as to where and how the production process is affected so that a modeled farmer can identify and apply the appropriate risk measures. Such information is more useful in annual planning decisions. ARS scores could be derived from 71 points, with an average score of less than 10 throughout the future time series for winter wheat in Kraichgau region (Table 3). This conclusion suggests that using near-term climate projections of a rather statistical nature, such as WETTREG 2010 [57], has a relatively low impact for winter wheat for a given average *sgy* threshold based on the Kraichgau regional sample. Considering that the average regional *sgy* threshold for this region is less than the region’s average yield (Table 1), the picture could be drawn completely differently for any of the 13 highly sensitive farmer types found in the empirical sample who declared their *sgy* threshold above 69.17 dt/ha. However, a more in-depth analysis is left for future research applying the ARS methodology.

### ARS scores in a dynamic context

Given a series of weather years where mid-season observations appear to be poor determinants of yield outcomes greater than the *sgy*, farmers gain new experience. When apparently risky inter-temporal states lead to learning processes that change the perception of crop response to climatic influence, the *sgy* approach can be used to provide the foundation of learning processes, thus creating a useful feature for bio-economic models.

The decision-making mechanism in FarmActor is designed to modify annual decisions to account for long-term trends in climate, with implied relationships between production risk and annual weather [32].As a consequence, the observation parameter value ranges can change over time.

#### How acceptance ranges can adapt

As a consequence of evaluating inter-temporal production outcomes, the following implications are possible for the perception mechanism in the simulation model: (1) no consequence and (2) a shift in the acceptance range. These implications can be the consequence of three possible scenarios. First, an observation value is within the acceptance range and a yield higher than or equal to the *sgy* is achieved; then, the entire process can be assessed as having gone as expected for a given parameter value. The observed value from the now past production year is included in the distribution of observation points that constitute the observation parameters’ acceptance ranges for the upcoming year without causing a shift of the range. Second, if an observed value is outside the acceptance range and a yield lower than the *sgy* is achieved, the observation parameter range for the upcoming year is not affected since this was an expected yield loss considering the set loss threshold (*sgy*). The third consequence is that observational fluctuations lie outside the acceptance range but do not result in negative consequences for the acceptance ranges because a yield above the *sgy* threshold is achieved. In that case, the observation is collated as a non-downside-risk value for the distribution of observations for a given parameter. However, depending on the amplitude of the fluctuation, a shift in the acceptance range may occur. When such outliers accumulate, they will add to a shift in the acceptance range that changes the expected performance of a given crop in its response to climatic (and management) influences. The larger the amplitude of the deviating value is, the longer it will most likely remain among the outliers that are excluded during the initialization process for the acceptance ranges in the model. Many outliers over several years in a row—again, depending on the learning mechanism—may indicate that a crop is quite difficult to cultivate at a given location. This can occur even though the simulated farmer annually adapts production procedures while also responding to daily environmental conditions.

### Use of ARS scores for comparative analyses

Additionally, different learning patterns that assign weights to ARS scores allow the study of the arrangement of a farmer’s crop portfolio and, thus, of land use over time. It is thus possible to give information about the climatic influence on crop portfolio and crop sequence choices as opposed to influences from prices and policies.

For crops that are not yet established in a region, simulation models could provide information regarding when cultivation of a certain crop in a region may become more likely and economically attractive. This analysis can be done by applying acceptance ranges governed by an averaged *sgy* level of a comparative region to growth processes of a potential future crop in the region of interest and under future climatic conditions.

## Conclusion

This work introduces a new approach for bio-economic simulation models to integrate farmer perceptions of climatic changes through an economic downside-risk evaluation of plant performance at various stages of production. The approach operates alongside the modeling of the production process, which underlies an optimization mechanism that governs the immediate decision-making process.

Developed at the field level for a small-scale study region, this methodology was designed to be customized for any site to study the perception processes of farmers. It can be applied with relatively little and cost-efficient empirical effort once a bio-economic simulation model has been calibrated for a study site. The methodology may find its way into other regions, for which bio-economic simulation models are used for analysis of strategic decision-making processes in crop production. The above approach may be particularly interesting for farming regions where major components of the crop yield are not brought to market or where market prices are not available; an economic risk analysis regarding climate change should still be conducted. The *sgy* approach does not require price modeling for an economic risk analysis because the economic valuation of profitability is embedded and expressed in yield or other biophysical terms.

### Recommendations

In the current version of the FarmActor model, the field allocation process is performed according to a predefined crop rotation that is based on a Markov sequence, relying on empirical observations of the past [60]. What occurs during the production cycle is no longer relevant at the time of the simulated field-use planning. Economic considerations are not incorporated in the field allocation process. The ARS score is the missing link to be used at the time of planning [61] for climate change impact analysis.

#### ARS scores in that context should be used as a constraint

The constraint should be modeled as follows:

$$\sum_{f}^{}\sum_{c}^{}ARS_{c}\geq ARS_{0,c}=\hat{ARS_{c}}$$ (3)

were *f* = field and *c* = crop;

*ARS*_0,*c*_ = the reference ARS score for the current year (the latest year for which observations are available) of all fields that have been allocated with a crop c [60].

${\hat{ARS}}_{c}$ = the expected ARS score for an activity (i.e., crop) as explained in the following. The ARS score for an activity should not be lower than the expected ARS score for an activity. Otherwise, certain fields should no longer be planted with a certain crop, or management options need to be evaluated in the model, which would provide an improvement in the production process.

The expected ARS score ($\hat{ARS_{c,t}}$) of crop c in year t is the average ARS score of past production processes for a certain crop in a farmer’s fields. How the average ARS score is calculated is subject to how learning is modeled and thus past observations are weighted, a topic left for future research. Learning depends on the weighting of past observations and if-then rules [24], which lead to consequences for the farm agent´s decision-making in bio-economic models.

Overall, the *sgy* approach is designed to provide a more comprehensive understanding of the drivers of farm planning decisions by explicitly modeling how farmers monitor production. Only when such underlying processes are better represented in more up-scaled bio-economic simulation models [62, 63] can deterministic statements about the climatic impact on agricultural production and land-use decisions be improved, as stated by White et al. [16].

Future research should aim to attach different valuations and learning patterns to this suggested mechanism and thus gain a more realistic and less assumption-driven understanding of farmers’ climate change perceptions and adaptation responses in their land-use decisions [62]. This approach may even assist in diminishing over- and underestimations of the rate of adaptation, which many models show through strong behavioral assumptions despite the high degree of complexity and level of detail of the approach [13–16, 33].

## Supporting information

S1 Questionnaire (PDF)

## References

[1] Foley JA, DeFries R, Asner GP, Barford C, Bonan G, Carpenter SR, et al Global Consequences of Land Use. Science. 2005; 309:570 doi: 10.1126/science.1111772 16040698 10.1126/science.1111772

[2] Högy P, Brunnbauer M, Koehler P, Schwadorf K, Breuer J, Franzaring J, et al Grain quality characteristics of spring wheat (Triticum aestivum) as affected by free-air CO2 enrichment. Environmental and Experimental Botany. 2013; 88(0): 11–8.

[3] Högy P, Fangmeier A. Effects of elevated atmospheric CO2 on grain quality of wheat. Journal of Cereal Science. 2008; 48(3): 580–91.

[4] Högy P, Poll C, Marhan S, Kandeler E, Fangmeier A. Impacts of temperature increase and change in precipitation pattern on crop yield and yield quality of barley. Food Chemistry. 2013; 136(3–4): 1470–7. doi: 10.1016/j.foodchem.2012.09.056 23194550 10.1016/j.foodchem.2012.09.056

[5] Nelson GC, van der Mensbrugghe D, Ahammad H, Blanc E, Calvin K, Hasegawa T, et al Agriculture and climate change in global scenarios: why don't the models agree. Agricultural Economics. 2014; 45(1): 85–101.

[6] Schmitz C, van Meijl H, Kyle P, Nelson GC, Fujimori S, Gurgel A, et al Land-use change trajectories up to 2050: insights from a global agro-economic model comparison. Agricultural Economics. 2014; 45(1): 69–84.

[7] IPCC. Climate Change 2014: Synthesis Report. Contribution of Working Groups I, II and III to the Fifth Assessment Report of the Intergovernmental Panel on Climate Change [Core Writing Team, Pachauri RK and Meyer LA (eds.)]. IPCC, Geneva, Switzerland; 2014: 151 pp.

[8] Crane TA, Roncoli C, Hoogenboom G. Adaptation to climate change and climate variability: The importance of understanding agriculture as performance. NJAS—Wageningen Journal of Life Sciences. 2011; 57(3–4): 179–85.

[9] Beddow JM, Pardey PG. Moving Matters: The Effect of Location on Crop Production. Journal of Economic History. 2015; 75(1): 219–49.

[10] Bloomfield JP, Williams RJ, Gooddy DC, Cape JN, Guha P. Impacts of climate change on the fate and behaviour of pesticides in surface and groundwater—a UK perspective. Science of the Total Environment. 2006; 369(1–3): 163–77. doi: 10.1016/j.scitotenv.2006.05.019 16914182 10.1016/j.scitotenv.2006.05.019

[11] Dury J, Schaller N, Garcia F, Reynaud A, Bergez J. Models to support cropping plan and crop rotation decisions. A review. Agronomy for Sustainable Development. 2012; 32(2): 567–80.

[12] Mußsshoff O, Hirschauer N. Modernes Agrar-Management. Betriebswirtschaftliche Analyse- und Planungsverfahren. Vahlen, München 2011.

[13] Just DR, Peterson HH. Diminishing Marginal Utility of Wealth and Calibration of Risk in Agriculture. American Journal of Agricultural Economics. 2003; 85(5): 1234–41.

[14] Just RE, Pope RD. Agricultural Risk Analysis: Adequacy of Models, Data, and Issues. American Journal of Agricultural Economics. 2003; 85(5): 1249–56.

[15] Ortiz-Bobea A, Just RE. Modeling the Structure of Adaptation in Climate Change Impact Assessment. American Journal of Agricultural Economics. 2013; 95(2): 244–51.

[16] White JW, Hoogenboom G, Kimball BA, Wall GW. Methodologies for simulating impacts of climate change on crop production. Field Crops Research. 2011; 124(3): 357–68.

[17] Henseler M, Wirsig A, Herrmann S, Krimly T, Dabbert S. Modeling the impact of global change on regional agricultural land use through an activity-based non-linear programming approach. Agricultural Systems. 2009; 100(1–3): 31–42.

[18] Lehmann N, Finger R, Klein T, Calanca P, Walter A. Adapting crop management practices to climate change: Modeling optimal solutions at the field scale. Agricultural Systems. 2013; 117(0): 55–65.

[19] Gbetibouo GA, 2009. Understanding Farmers´ Perceptions and Adaptations to Climate Change and Variability. The Case of the Limpopo Basin, South Africa. IFPRI Discussion Paper 00849. IFPRI Environment and Production Technology Division. [Internet] 2009. [cited 2017 June 13] Available from: http://www.fao.org/fileadmin/user_upload/rome2007/docs/ifpri_limpopo_dp00849.pdf.

[20] Schönhart M, Schauppenlehner T, Schmid E, Muhar A. Integration of bio-physical and economic models to analyze management intensity and landscape structure effects at farm and landscape level. Agricultural Systems 2011; 104 (2011):122–34.

[21] Troost C, Berger T. Dealing with Uncertainty in Agent-Based Simulation: Farm-Level Modeling of Adaptation to Climate Change in Southwest Germany. American Journal of Agricultural Economics. 2014 8 31.

[22] Williams JR, Jones CA, Kiniry JR, Spanel DA. The EPIC Crop Growth Model. Transactions of the ASAE. 1989; 32(2): 0497–0511.

[23] Priesack E, Gayler S, Hartmann H. The impact of crop growth sub-model choice on simulated water and nitrogen balances. Nutrient Cycling in Agroecosystems. 2006; 75: 1–13.

[24] McCown RL, Carberry PS, Dalgliesh NP, Foale MA, Hochman Z. Farmers use intuition to reinvent analytic decision support for managing seasonal climatic variability. Agricultural Systems. 2012; 106(1): 33–45.

[25] Antle JM. Asymmetry, Partial Moments, and Production Risk. American Journal of Agricultural Economics. 2010 10 07.

[26] Finger R. Biases in farm-level yield risk analysis due to data aggregation. German Journal of Agricultural Economics. 2012; 61(1): 30–43.

[27] Groom B, Koundouri P, Nauges C, Thomas A. The story of the moment: risk averse cypriot farmers respond to drought management. Appl Econ. 2008; 40(3): 315–26.

[28] Lehmann N, Briner S, Finger R. The impact of climate and price risks on agricultural land use and crop management decisions. Land Use Policy. 2013; 35: 119–30.

[29] Mirzaeitalarposhti R, Demyan MS, Rasche F, Cadisch G, Müller T. Mid-infrared spectroscopy to support regional-scale digital soil mapping on selected croplands of South-West Germany. Catena. 2017; 149, Part 1: 283–93.

[30] Schreinemachers P, Berger T. An agent-based simulation model of human–environment interactions in agricultural systems. Environmental Modelling & Software 2011; 26: 845–59.

[31] Keating BA, Carberry PS, Hammer GL, Probert ME, Robertson MJ, Holzworth D, et al An overview of APSIM, a model designed for farming systems simulation. European Journal of Agronomy. 2003 1; 18(3–4): 267–88.

[32] Aurbacher J, Parker PS, Calberto Sánchez GA, Steinbach J, Reinmuth E, Ingwersen J, et al Influence of climate change on short term management of field crops–A modelling approach. Agricultural Systems. 2013; 119(0): 44–57.

[33] McCown RL, Wafula BM, Mohammed L, Ryan JG, Hargreaves JNG. Assessing the value of a seasonal rainfall predictor to agronomic decisions: the case of response farming in Kenya In: Muchow RC, Bellamy JA, editors. Climatic Risk in Crop Production: Models and Management for the Semi Arid Tropics and Subtropics. CAB International: Wallingford, UK; 1991 p. 383–409.

[34] Biernath C, Bittner S, Klein C, Gayler S, Hentschel R, Hoffmann P, et al Modeling acclimation of leaf photosynthesis to atmospheric CO2 enrichment. European Journal of Agronomy. 2013; 48(0): 74–87.

[35] Biernath C, Gayler S, Bittner S, Klein C, Högy P, Fangmeier A, et al Evaluating the ability of four crop models to predict different environmental impacts on spring wheat grown in open-top chambers. European Journal of Agrononmy. 2011; 35(2): 71–82.

[36] Godwin D, Ritchie J, Singh U, Hunt L. A User’s Guide to CERES-Wheat- V2.10. AL, USA: International Fertilizer Development Center, Muscle Shoals; [Internet] 1990 [cited 2017 June 13] Available from: http://pdf.usaid.gov/pdf_docs/PNABU270.pdf.

[37] Ritchie JT, Wheat Phasis Development, Modeling Plant and Soil Systems. Madison, editor. WI: USA; 1991: p. 31–54.

[38] Parker P, Ingwersen J, Högy P, Priesack E, Aurbacher J. Simulating regional climate-adaptive field cropping with fuzzy logic management rules and genetic advance. The Journal of Agricultural Science. 2015: 1–16.

[39] Parker PS, Shonkwiler JS, Aurbacher J. Cause and Consequence in Maize Planting Dates in Germany. Journal of Agronomy and Crop Science. 2017; (203):227–40.

[40] Ingwersen J, Steffens K, Högy P, Warrach-Sagi K, Zhunusbayeva D, Poltoradnev M, et al Comparison of Noah simulations with eddy covariance and soil water measurements at a winter wheat stand. Agriculture and Forest Meteorolgy. 2011; 151(3): 345–55.

[41] Wizemann HD, Ingwersen J, Högy P, Warrach-Sagi K, Streck T, Wulfmeyer V. Three-year observations of water vapor and energy fluxes over agricultural crops in two regional climates of Southwest Germany. Meteorologische Zeitschrift 2015; 24: 39–59.

[42] Dorward AR, Parton KA. Quantitative farm models and embedded risk in complex, diverse and risk prone agriculture. Quarterly Journal of International Agriculture. 1997; 36(4): 317–30.

[43] Ewert F, Rötter RP, Bindi M, Webber H, Trnka M, Kersebaum KC, et al Crop modelling for integrated assessment of risk to food production from climate change. Environmental Modelling & Software. 2015; 72: 287–303.

[44] Just DR, Khantachavana SV, Just ER. Empirical Challengens for Risk Preferences and Production. Annual Review of Resource Economics. 2010; 2: 13–31.

[45] Hardaker JB, Huirne RB, Anderson JR, Lien G. Coping with risk in agriculture 2nd ed. CABI publishing; 2004.

[46] Finger R. Expanding risk consideration in integrated models–The role of downside risk aversion in irrigation decisions. Environmental Modelling & Software. 2013; 43(0): 169–72.

[47] Arrow KJ. The Role of Securities in the Optimal Allocation of Risk-bearing. The Review of Economic Studies. 1964; 31(2): 91–6.

[48] Pratt JW. Risk Aversion in the Small and in the Large. Econometrica. 1964; 32(1/2): 122–36.

[49] Kellner U, Mußhoff O, Battermann H. The Economic Valuation of Irrigation under Consideration of risk and Changes in water Withdrawal Permits. German Journal of Agricultural Economics. 2012; 61(1).

[50] LEL Strukturdaten zur Landwirtschaft in Baden-Württemberg [dataset on the Internet] 2015. [cited 2017 June 13]. Available from: https://www.google.de/url?sa=t&rct=j&q=&esrc=s&source=web&cd=1&ved=0ahUKEwiw0vvyqonNAhUDQBQKHWapD-IQFggkMAA&url=http%3A%2F%2Fwww.landwirtschaft-bw.info%2Fpb%2Fsite%2Flel%2Fget%2Fdocuments%2FMLR.LEL%2FPB5Documents%2Flel%2FAbteilung_3%2FAgrarstuktur%2FStrukturdaten%2FInteraktive%2520Daten%2FStrukturdaten%2520Baden-W%25C3%25BCrttemberg.xls%3Fattachment%3Dtrue&usg=AFQjCNEWab70FbdBZcFRagxefCAI_9FrAg&cad=rja.

[51] Flichman G, Allen T. Bio-economic modeling: State-of-the-art and key priorities International Food Policy Research Institute (IFPRI) Washington, D.C. [Internet] 2014 [cited 2017 June 13]. Available from: http://ebrary.ifpri.org/cdm/ref/collection/p15738coll2/id/129231.

[52] Hardaker JB, Pandey S, Patten LH. Farm planning under uncertainty: a review of alternative programming models. Review of Marketing and Agricultural Economics. 1991;59(01): 9–22.

[53] Kahnemann D. Experienced Utility and Objective Happiness: A Moment-Based Approach In: Kahnemann D, Tversky A, editors. Choices, Values and Frames. New York: Cambridge University Press and the Russell Sage Foundation; 2000: p. 673–692.

[54] Dorward A. Modelling embedded risk in peasant agriculture: methodological insights from northern Malawi. Agricultural Economics. 1999; 21(2): 191–203.

[55] JKI. Growth stages of mono- and dicttyledonomous plants. BBCH Monograph. 2nd Edition. Meier U, editor. Federal Biological Research Centre for Agriculture and Forestry (JKI), editor. [Internet] 2001 [cited 2017 June 13] Available from: http://pub.jki.bund.de/index.php/BBCH/issue/view/161. 2001.

[56] StataCorp. Stata Statistical Software: Release 13. College Station, TX: StataCorp LP [software]. 2013.

[57] Kreienkamp F, Enke W, Spekat A. WR2010_EH5_1_A1B: UBA-WETTREG ECHAM5/OM 20C + A1B Lauf 1 realization run 1961–2100 [dataset]. World Data Center for Climate. CERA-DB ‘‘WR2010_EH5_1_A1B”. 2010 [cited 2017 June 13] Available from: http://www.dwd.de/DE/leistungen/deutscherklimaatlas/erlaeuterungen/klimaszenarien/klimaszenarien_node.html.

[58] DWD Climate Data Center (CDC) [dataset]. Historical daily station observations (temperature, pressure, precipitation, wind, sunshine duration, etc.) for Germany, version v004, 2016. Available from: ftp://ftp-cdc.dwd.de/pub/CDC/observations_germany/climate/daily/kl/historical/.

[59] IPCC. Climate Change 2007: Synthesis Report. Contribution of Working Groups I, II and III to the Fourth Assessment Report of the Intergovernmental Panel on Climate Change [Core Writing Team, Pachauri, R.K and Reisinger, A. (editors.)]. IPCC, Geneva: Switzerland. [Internet] 2007 [cited 2017 June 13]: [104 pp.] Available from: http://www.ipcc.ch/publications_and_data/ar4/syr/en/frontmatter.html.

[60] Aurbacher J, Dabbert S. Generating crop sequences in land-use models using maximum entropy and Markov chains. Agricultural Systems. 2011; 104(6): 470–9.

[61] Reinmuth E, Dabbert S. Toward more efficient model development for farming systems research–An integrative review. Computers and Electronics in Agriculture 2017;138:29–38.

[62] Berger T, Troost C. Agent-based Modelling of Climate Adaptation and Mitigation Options in Agriculture. Journal of Agricultural Economics. 2014; 65(2): 323–48.

[63] Stöckle CO, Donatelli M, Nelson R. CropSyst, a cropping systems simulation model. European Journal of Agronomy. 2003; 18(3–4): 289–307.

