# Supplementary material for: Modeling perceptions of climatic risk in crop production
Source: PLoS One. 2017 Aug 1;12(8):e0181954. doi: 10.1371/journal.pone.0181954 (PMC5538739; doi:10.1371/journal.pone.0181954)
Supplement: S1 Questionnaire — (PDF) [file pone.0181954.s001.pdf]

Questionnaire Regional Climate Change

Dear Sir or Madam,
Thank you for participating in our data collection survey. Data assessed in this questionnaire will be analyzed for scientific purposes only and treated with the safeguard of anonymity and confidentiality within the framework of the DFG (German Research Foundation) research project "regional climate change".

1. Questions regarding your farming operation

1.1 Where is your farm located? postal code ______ region (Kraichgau/Swabian Alb): ______

1.2 How do you manage your operation? main source of income ☐ secondary source of income ☐

1.3 Is it a family farm? yes ☐ no ☐

1.4 What form of organization does your farm have?
(e.g. GbR (German equivalent for: company consituted under civil law)) ______

1.5 Which production method to you apply? a) conventional farming ☐ c) converting to organic farming ☐
b) organic farming ☐

1.6 If b) or c): Are you a member of a growers association? yes ☐ no ☐

1.7 What is your main operation focus/ what are your operation focuses? *In case of several focuses, please order them and list your primary focus first.*

1.8 In case of animal husbandry, please list the number of animals kept:

dairy cows	☐	beef cows/ bulls	☐	laying hens	☐
suckler cows	☐	breeding sows	☐	fattening poultry	☐
offspring	☐	fattening pigs	☐	others:	______

1.9 How many hectares do you manage?

pasture	______	ha	lease ratio	______	ha
arable land	______	ha	lease ratio	______	ha
special cultivation	______	ha	lease ratio	______	ha
forest	______	ha	lease ratio	______	ha

1.10 What crop rotation do you practice? Do you have several crop rotations?

	crop rotation	In case of differing crop rotations, what is the reason? And how do the location characteristics differ?
1st crop rotation:		
2nd crop rotation:		
3rd crop rotation:		

1.11 Please name all crops/cultivars you usually grow on your farm with their characteristics. Please also list fodder crops like clover grass, for example:

						yield levels according to your experience on your farm area			
cultivar and (optional) quality grade	winter or summer crop	cultivated area (ha)	sale (%)	on farm consumption (%)	type of usage (e.g. baking, fodder, energy corn)	peak yield in dt/ha	"still-good" yield in dt/ha	average yield in dt/ha	reference yield level for fertilization

1.12 Did you sell or buy products over the last three years that are connected to bio-energy? If yes, please list:

year	silage corn		whole crop silage		fermentation residues		manure		others:	
	amount (dt)	sold/bought	amount (dt)	sold/bought	amount (m³)	sold/bought	amount (m³)	sold/bought	amount	sold/bought
2010										
2011										
2012										

1.13 Which part of the growth development, according to your experience and at your location, is especially risky in crop production? What kind of risks occur and how do you deal with them? Please refer to your most important field crop:

growth stage	period	risk	What characteristics do you use to assess the quality of the area under crop at that stage?	How do you react to a deviation from the desired growth development stage?	probable yield losses (%)
e.g. grain filling	*e.g. August/September*	*e.g. heat/drought*	*e.g. degeneration…*	*e.g. irrigation/ no reaction possible*	*1-100 %*
**Please name your most important crop: ___________________________________**					

[…

The part of this questionnaire that was excluded contains questions that do not concern the content of this paper and are thus left out.

…]

Do you have further remarks or comments for us?

I want to participate in the lottery drawing with the chance to win a BayWa (local farm supply company) voucher. My address is:

In order to make this project a great success and a win-win situation for everyone involved, including you, we believe it is very important for farmers and scientists to cooperate and communicate. Would you like to be invited to events like workshops or meetings? If so, please provide us with your contact information:

- ☐ As mentioned above for the lottery drawing
- ☐ Please contact me using the following address/e-mail address:

Thank you for your participation and the time you took to respond to this questionnaire!
